# Pronounced expression of the lipolytic inhibitor G0/G1 Switch Gene 2 (G0S2) in adipose tissue from brown bears (*Ursus arctos*) prior to hibernation

**DOI:** 10.14814/phy2.12781

**Published:** 2016-04-25

**Authors:** Niels Jessen, Thomas S. Nielsen, Mikkel H. Vendelbo, Rikke Viggers, Ole‐Gunnar Støen, Alina Evans, Ole Frøbert

**Affiliations:** ^1^ Research Laboratory for Biochemical PathologyInstitute for Clinical MedicineAarhus UniversityAarhusDenmark; ^2^ The Novo Nordisk Foundation Center for Basic Metabolic ResearchFaculty of Health and Medical SciencesUniversity of CopenhagenCopenhagenDenmark; ^3^ Department of Nuclear Medicine and PET CenterAarhus University HospitalAarhusDenmark; ^4^ Department of Ecology and Natural Resource ManagementNorwegian University of Life SciencesAasNorway; ^5^ Department of Forestry and Wildlife ManagementFaculty of Applied Ecology and Agricultural SciencesHedmark University CollegeCampus EvenstadKoppangNorway; ^6^ Faculty of HealthDepartment of CardiologyÖrebro UniversityÖrebroSweden

**Keywords:** Adipose tissue, adipose tissue triglyceride lipase, fatty acid/metabolism, lipolysis

## Abstract

Prior to hibernation, the brown bear (*Ursus arctos*) exhibits unparalleled weight gain. Unlike humans, weight gain in bears is associated with lower levels of circulating free fatty acids (FFA) and increased insulin sensitivity. Understanding how free‐ranging brown bears suppress lipolysis when gaining weight may therefore provide novel insight toward the development of human therapies. Blood and subcutaneous adipose tissue were collected from immobilized free‐ranging brown bears (fitted with GPS‐collars) during hibernation in winter and from the same bears during the active period in summer in Dalarna, Sweden. The expression of lipid droplet‐associated proteins in adipose tissue was examined under the hypothesis that bears suppress lipolysis during summer while gaining weight by increased expression of negative regulators of lipolysis. Adipose triglyceride lipase (ATGL) expression did not differ between seasons, but in contrast, the expression of ATGL coactivator Comparative gene identification‐58 (CGI‐58) was lower in summer. In addition, the expression of the negative regulators of lipolysis, G0S2 and *c*ell‐death *i*nducing *D*NA fragmentation factor‐a‐like *e*ffector (CIDE)C markedly increased during summer. Free‐ranging brown bears display potent upregulation of inhibitors of lipolysis in adipose tissue during summer. This is a potential mechanism for increased insulin sensitivity during weight gain and G0S2 may serve as a target to modulate insulin sensitivity.

## Introduction

The current global pandemic of obesity and associated diseases pose a major challenge for the wellbeing of the affected individuals and for health care providers. Obesity is closely associated with insulin resistance and this condition is a fundamental aspect of the etiology of type 2 diabetes (Kahn and Flier 2000). Insulin resistance precedes the onset of diabetes by 1–2 decades (Warram et al. 1990) and lifestyle interventions that increase insulin sensitivity prevent the onset of diabetes in high‐risk populations (Pan et al. 1997; Tuomilehto et al. 2001; Knowler et al. 2002). However, few pharmacologic approaches to increase insulin sensitivity have been successful and novel strategies are needed and inspiration for such may come from observing hibernating brown bears (*Ursus arctos*). During winter these animals are physical inactive and bradycardic but do not develop vascular or heart disease. Unlike most other hibernators, brown bears only reduce body temperature marginally during hibernation. Data suggest that bears have developed mechanisms for healthy weight gain. Prior to hibernation, the brown bear exhibits hyperphagia and weight gain unparalleled by humans (Swenson et al. 2007). However, unlike what is observed is humans, weight gain in brown bears is associated with lower levels of free fatty acids (FFA) (Graesli et al. 2015). By investigating brown bears living in captivity it has been shown that insulin sensitivity is increased prior to hibernation (Professor L. Nelson, pers. comm. 2016, Washington State University, USA). FFAs are released by hydrolysis of triglycerides in adipose tissue during lipolysis and inappropriate stimulation of lipolysis, as seen with obesity, has been suggested to play a major role in the underlying pathophysiology of type 2 diabetes (Boden and Shulman 2002). Understanding how brown bears suppress lipolysis during summer when gaining weight may therefore provide valuable insight toward the development of human therapies.

In the adipocyte triglycerides are hydrolyzed to free fatty acids (FFA) and glycerol by the sequential action of Adipose Triglyceride Lipase (ATGL), Hormone Sensitive Lipase (HSL) and Monoglyceride Lipase. ATGL catalyzes the rate limiting step in lipolysis (Zimmermann et al. 2004), and the enzyme activity is tightly regulated by complex formation between ATGL and several lipid droplet‐associated proteins. ATGL activity is acutely increased via interaction with the coactivator comparative gene identification‐58 (CGI‐58) (Lass et al. 2006). In the basal state, CGI‐58 is complexed with lipid‐droplet‐coating protein perilipin 1 (PLIN1) and ATGL activity is low. Upon stimulation of lipolysis, for example, through adrenergic receptor signaling, PLIN1 is phosphorylated (Greenberg et al. 1991) and this promotes the release of CGI‐58 which binds to, and activates ATGL. The degree of stimulation of ATGL activity by CGI‐58 can be modulated by binding of the protein product of G0/G1 switch gene 2 (G0S2) to ATGL (Yang et al. 2010). Binding of G0S2 to ATGL appears to be dominant to activation by CGI‐58 (Yang et al. 2010) and the inhibition of ATGL by G0S2 is dose‐dependent (Schweiger et al. 2012). There is also evidence to suggest that the proapoptotic protein family termed *c*ell‐death *i*nducing *D*NA fragmentation factor‐a‐like *e*ffector (CIDE) may regulate ATGL activity. Two CIDE proteins, CIDE‐A and CIDE‐C, act as negative regulators of lipolysis, most likely by shielding of the lipid droplets from the action of lipases (Nordstrom et al. 2005; Puri et al. 2008) or by direct binding to ATGL (Grahn et al. 2014).

The aim of this study was to investigate the expression of lipid droplet associated proteins in adipose tissue from free‐ranging brown bears during hibernation in winter and during their active period in summer. The underlying hypothesis is that brown bears suppress lipolysis during summer when gaining weight by increased expression of negative regulators of ATGL activity.

## Materials and Methods

### Animal capture and biopsy procedure

Blood and subcutaneous adipose tissue were collected from immobilized free‐ranging brown bears (fitted with GPS‐collars) during hibernation in winter (early February) and, from the same bears, during the active period in early summer (late June/early July) in Dalarna, Sweden. All captures were approved by the Swedish Ethical Committee on Animal Research (C212/9 and C47/9) and the Swedish Environmental Protection Agency and follow the general principles of Public Health Service Policy on Humane Care and Use of Laboratory Animals applicable to wildlife. Sampling was done by the Scandinavian Brown Bear Research Project (http://www.bearproject.info/) according to established protocols (Arnemo et al. 2012).

### Blood analysis

Serum FFAs were analyzed with a commercially available kit (intraassay CV 2–4% and interassay CV 3–6%, detection limit 0.02 mmol/L) (NEFA‐HR 2; Wako Chemicals, Richmond, VA).

### Histology

Adipose tissue biopsies were fixed in 4% formalin buffer (Cellpath, Newtown, UK) and embedded in paraffin. 3 *μ*m sections were heated at 60°C for 60 min, deparaffinized in Tissue‐Clear (Sakura Finetek Europe, Alphan, the Netherlands) and rehydrated.

### Western blot analysis

Adipose biopsies (~100 mg) were homogenized in a Precellys 24 homogenizer (Bertin Technologies, Montigny‐le‐Bretonneux, France) in a hypotonic buffer containing 20 mmol/L HEPES pH 7.4, 10 mmol/L NaF, 1 mmol/L Na_3_VO_4_, 1 mmol/L EDTA, 5% SDS, 50 *μ*g/mL Soybean Trypsin Inhibitor, 4 *μ*g/mL Leupeptin, 0.1 mmol/L Benzamidine, 2 *μ*g/mL Antipain, and 1 *μ*g/mL Pepstatin. Crude homogenates were incubated in a Thermomixer (Eppendorf, Hamburg, Germany) at 37°C and 1000 rpm for 1 h followed by centrifugation at 14.000 × *g* for 20 min. The lipid‐free infranatant was aspirated and used for western blot analysis.

Protein samples were resolved by SDS‐PAGE (4–12% Bis‐Tris gels, Criterion XT system; Bio‐Rad, Hercules, CA) and transferred to polyvinylidene fluoride membranes according to manufacturer's instructions. The following primary antibodies were used: anti‐ATGL, anti‐CGI‐58 (Abcam, Cambridge, UK), anti‐PLIN1, anti‐HSL (Cell Signaling, Beverly, MA), anti‐CIDE‐C (Novus Biologicals, Cambridge, UK), and anti‐G0S2, anti‐uncoupling protein 1(Santa Cruz Biotechnology; Santa Cruz, CA). Horseradish peroxidase‐conjugated donkey anti‐rabbit IgG (Amersham, GE‐Healthcare, Pittsburgh, PA) and goat anti‐mouse HRP‐IgG (Abcam, Cambridge, UK) were used as secondary antibodies. Proteins were visualized by chemiluminescence (Pierce Supersignal West Dura, Thermo Scientific, IL) and quantified with the ChemiDoc^™^ MP imaging system (BioRad). Precision Plus All Blue protein standards were used as a marker of molecular weight (BioRad). Control for protein loading was performed using the Stain‐Free technology (Gurtler et al. 2013), and data are expressed as a ratio to total protein content in the membranes.

### Statistics

Normal distribution was tested by evaluation of QQ‐plots and histograms. When possible, nonparametric data were transformed to a normal distribution by means of a logarithmic transformation. Differences in G0S2 expression were evaluated using Fisher's exact test. Data are presented as mean ± SEM unless otherwise stated. Statistical analysis was performed by comparing hibernating and nonhibernating conditions within the same bear (paired *t*‐test) and *P* < 0.05 was regarded as statistically significant. Data were analyzed and graphs were designed in SigmaPlot (SigmaPlot 11.0, Systat Software, CA).

## Results

### Animal characteristics

The basic characteristics of the animals are summarized in Table 1. The bears gained weight between the two sampling periods, but the increase was only borderline significant (*P* < 0.08).

**Table 1:** Animals characteristics from five bears captured during winter and summer 2013

	Summer	Winter
Males/females	2/3	2/3
Age (years)	3	3
Weight (kg)	58 (38–64.5)	53 (40–55)*

Weight is expressed as median and range (*P < 0.08 vs. summer).

### Adipose tissue morphology

**Figure 1:**
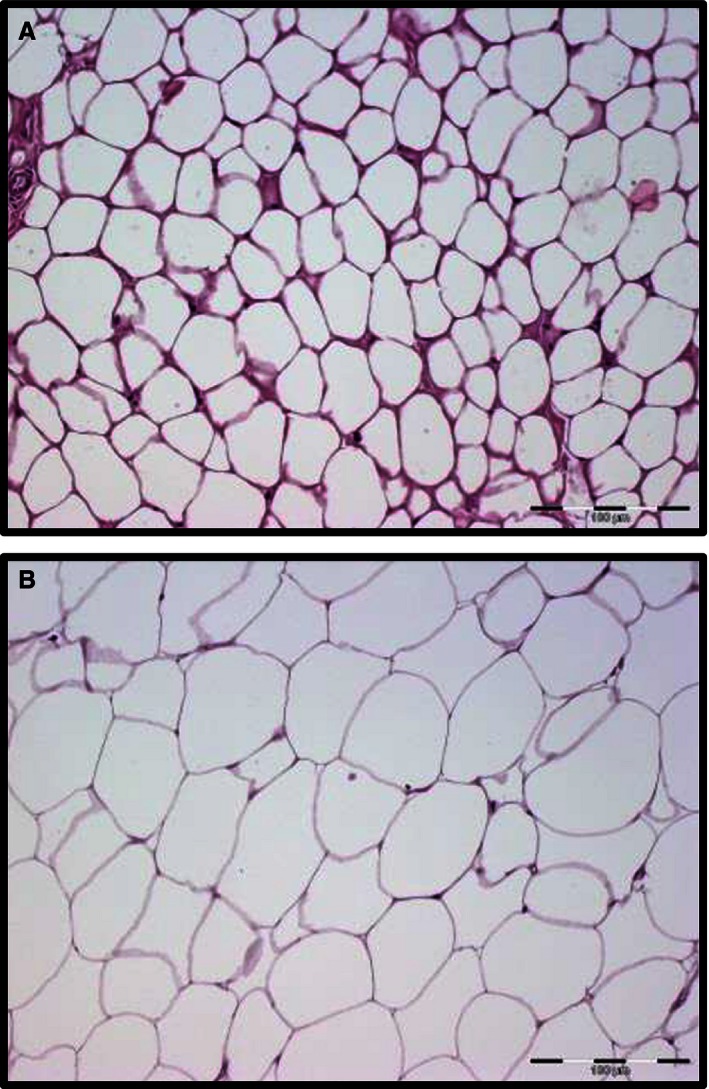
Hematoxylin and eosin staining of adipose tissue biopsies taken from the inguinal fat pad from the same immobilized free‐ranging brown bear (fitted with GPS‐collars) during summer (A) and during hibernation in winter (B) from Dalarna, Sweden.

As shown in Figure 1, adipocyte cell size was smaller during summer captures (A) than winter (B). Classical characteristics of white adipose tissue with monovacuolar cells containing a large lipid droplet surrounded by a layer of cytoplasm could be recognized during both seasons. As expected, we did not observe plurivacuolar cells characteristic of brown fat in any biopsies and no expression of uncoupling protein 1 was detected in the biopsies (data not shown).

### Blood samples

Circulating levels of FFA varied greatly among bears. Mean level during summer was 0.20 mmol/L (±0.08) and 0.43 mmol/L (±0.09) during winter, but the differences did not reach statistical significance.

### Inhibitors of lipolysis is increased in the nonhibernating condition

**Figure 2:**
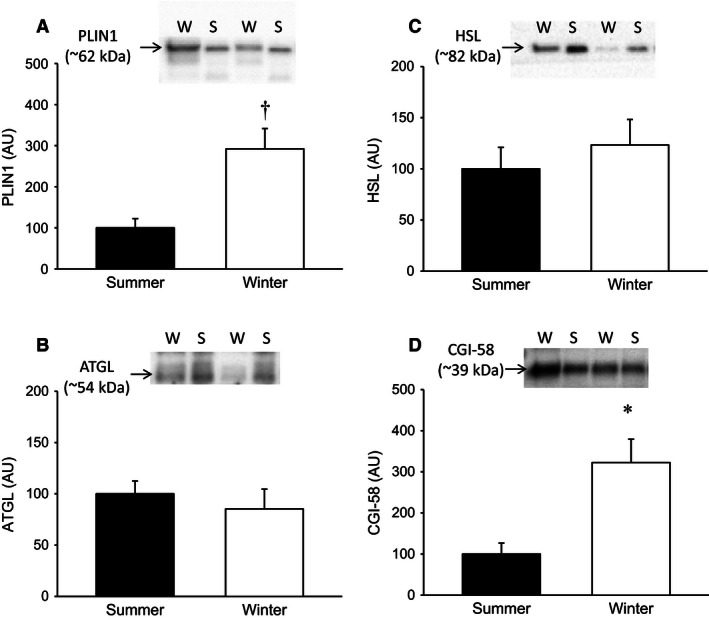
(A–B) Protein content of the prolipolytic cofactor CGI‐58 in adipose tissue biopsies during summer and winter (*P < 0.05 vs. summer, *N* = 5) and the lipid‐droplet‐coating protein perilipin expressed as a ratio of total protein content in the biopsy (^†^ *P* < 0.01 vs. summer, *N* = 5). (C–D) Protein levels of the lipases ATGL and HSL. (AU, Arbitrary units).

The expression of the prolipolytic cofactor CGI‐58 was during winter ~300% compared to summer levels (*P* < 0.05) (Fig. 2A). The expression of the adipocyte‐specific lipid‐coating protein PLIN1 was detected in both hibernating and nonhibernating conditions with lower expression levels in the adipose tissue taken during the summer compared to winter biopsies (Fig. 2B). Adipose tissue expression of the lipases ATGL and HSL (Fig. 2C and D) did not change during seasons.

**Figure 3:**
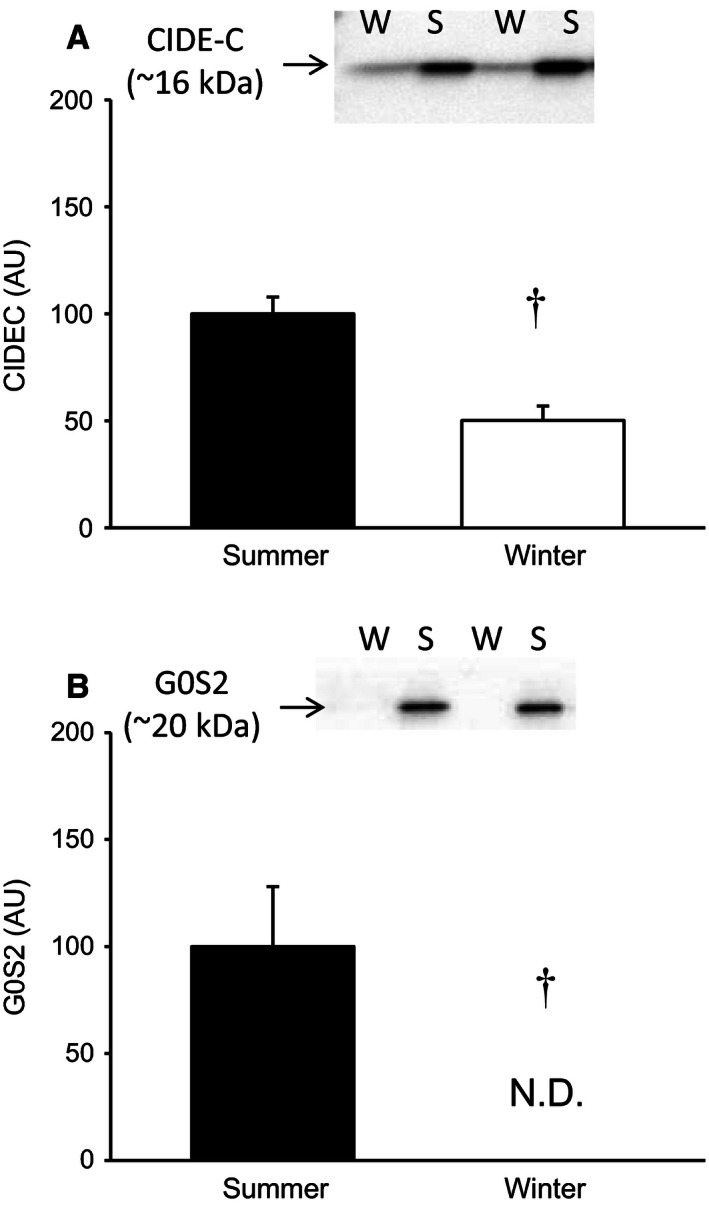
(A) Expression of CIDE‐C expressed as a ratio of total protein content in the biopsy (^†^ *P* < 0.01 vs. summer, *N* = 5). (B) The expression of the negative regulator of lipolysis G0S2 was completely ablated during hibernating conditions (N.D., Not Detected; AU, Arbitrary units; ^†^ *P* < 0.01, *N* = 5).

The expression of the negative regulators of lipolysis, G0S2 and CIDE‐C is depicted in Figure 3A and B. We found decreased expression of CIDE‐C (~50%) during winter (*P* < 0.01), whereas the expression of G0S2 was completely absent in adipose tissue from hibernating bears (*P* < 0.01).

## Discussion

By exploring the amazing adaptation for hibernation in free‐ranging brown bears we have found evidence for a molecular mechanism that can contribute to explaining the paradox of increased insulin sensitivity during weight gain that is observed in brown bears living in captivity. We suggest that the profound upregulation of inhibitors of lipolysis during summer inhibits release of FFA to the circulation during conditions characterized by a positive energy balance (Swenson et al. 2007). The inhibitory effects of elevating plasma FFA levels on peripheral insulin‐stimulated glucose uptake and oxidation are well‐established (Belfort et al. 2005; Gormsen et al. 2007; Hoeg et al. 2011), and the dynamic regulation of lipid‐droplet‐coating proteins in brown bears adds evidence to the central role of the adipocyte in control of whole body metabolism.

Several lines of evidence demonstrate that alterations in the expression of lipid‐droplet‐coating proteins play a prominent role in regulation of lipolysis. Ablation of G0S2 and CIDE‐C in rodents increases lipolytic rates in adipocytes (Nishino et al. 2008; Zhang et al. 2014). Under physiological conditions, prolonged fasting decreases the expression of G0S2 in humans and this is associated with a reduced insulin‐stimulated suppression of lipolysis (Nielsen et al. 2011). Similar observations have been made in fasting chicken, quails, and pigs (Oh et al. 2011; Ahn et al. 2013). This demonstrates that the mechanism is preserved among species, but the dramatic increase in the expression of G0S2 in brown bears during summer supersedes previously observed levels of regulation. Overexpression of ATGL in mice increases lipolytic activity (Ahmadian et al. 2009), but the inhibitory effects of G0S2 and CIDE‐C during summer was not compensated by elevations in ATGL expression. In fact, ATGL is only lipolytic active when associated with CGI‐58, and the increased lipolytic activity in ATGL overexpressing mice is associated with a 3.5‐fold increased level of CGI‐58 (Ahmadian et al. 2009). We found that CGI‐58 was markedly increased during hibernation and it is therefore likely that the substantial upregulation of the lipolytic inhibitors G0S2 and CIDE‐C in summer was potentiated by downregulation of CGI‐58 expression.

The expression levels of lipid‐coating proteins are likely to have clinical impact for patients with type 2 diabetes. CIDE‐C expression is reduced in obese subjects on a very low calorie diet (Magnusson et al. 2008), and this will likely contribute to weight loss and ultimately an improvement in metabolic regulation. However, reduced expression of inhibitors of lipolysis can also have negative clinical consequences. Poor glycemic control in diabetic patients is associated with reduced G0S2 expression in adipose tissue and elevated circulating FFA levels (Nielsen et al. 2012). This can initiate a viscous cycle with increased insulin resistance in skeletal muscle and further dysregulation of glycemic control (Boden and Shulman 2002). In humans, treatment with thiazolidinediones inhibits lipolysis and this is associated with increased insulin sensitivity and a favorable endocrine activity of adipose tissue (DeFronzo 2009). However, due to undesirable off‐target effects of thiazolidinediones this treatment is not recommended for most diabetics, and other strategies to inhibit lipolysis are warranted. Compounds that mimic the inhibitory action of CIDES and G0S2 could therefore be of clinical relevance. It is therefore interesting that a recent study has demonstrated that a synthetic peptide corresponding to a short sequence of G0S2 can inhibit ATGL activity in the nanomolar range (Cerk et al. 2014). If such compounds can be utilized in a clinical setting, it may be possible for humans to mimic some of the metabolic adaptations observed in brown bears.

Studying free‐ranging wildlife poses obvious limits for which measurements are feasible to perform. Investigating the bears in late summer during hyperphagic conditions is not feasible as this can interrupt the animals preparation to hibernating phase. We did investigate lipolytic signaling to hormone sensitive lipase, but observed great variation among samples. This is likely an artifact explained by differences in conditions of the individual captures resulting in variable catecholamine levels. Although less pronounced, we also observed variation in circulating FFA levels between bears and this measurement may also be influenced by the captures. However, the seasonal levels we measured in free‐ranging brown bears correspond to observations in brown bears living in captivity (Professor Lynne Nelson, Washington State University USA, personal communication), and the fact that the seasonal difference in FFA levels did not reach statistical significance is due to low statistical power. A previous report on seasonal changes in serum chemistry in a larger group of free‐living bears found significant differences between winter and summer, but not between winter and spring (Graesli et al. 2015). Unlike catecholamine signaling and FFA levels, the expression levels of lipid‐coating proteins are unlikely to be significantly affected by the conditions of the capture because the bears are usually anesthetized within minutes, and although the half‐life of G0S2 is short (<1 h [Yang et al. 2011]), it is unlikely that protein expression levels change over such short time frame.

In conclusion, free‐ranging brown bears display potent upregulation of inhibitors of lipolysis in adipose tissue during summer. This may serve as an underlying mechanism for the increased insulin sensitivity during weight gain previously observed in bears living in captivity. Accordingly, G0S2 may serve as a target to modulate whole body glucose homeostasis.

## Conflict of Interest

None declared.
